# Patient experience of alpha-1 antitrypsin deficiency-associated liver disease: a qualitative study

**DOI:** 10.1007/s11136-025-03926-x

**Published:** 2025-03-13

**Authors:** Virginia C. Clark, Suna Park, Robert Krupnick, Nicole Sparling, Jason Ritchie, Chitra Karki, Justin A. Reynolds

**Affiliations:** 1 University of Florida, Gainesville, FL USA; 2 Takeda Development Center Americas, Inc., Cambridge, MA USA; 3 IQVIA Patient Centered Solutions, Boston, MA USA; 4 IQVIA Patient Centered Solutions, New York, NY USA; 5 St. Joseph’s Hospital and Medical Center, Phoenix, AZ USA; 6 Creighton University School of Medicine, Phoenix, AZ USA

**Keywords:** Alpha-1 antitrypsin deficiency, Conceptual model, Disease impact, Genetic liver disease, Liver disease symptoms, Patient experience

## Abstract

**Purpose:**

To elicit the signs and/or symptoms, and impacts on daily living experienced by patients with alpha-1 antitrypsin deficiency-associated liver disease (AATD-LD).

**Methods:**

A preliminary “concept list” of signs and/or symptoms, and impacts was developed from a targeted literature review, patient blog posts, and clinician interviews. Subsequently, one-to-one concept elicitation interviews involving English-speaking, US adults with AATD-LD and a protease inhibitor (Pi) ZZ or MZ genotype were conducted by trained interviewers following a central Institutional Review Board-approved discussion guide. An AATD-LD conceptual model was developed based on these findings. Concepts were “most salient” if reported by ≥ 8 patients with a mean bothersomeness/disturbance rating of ≥ 5, or “highly salient” if reported by > 5– < 8 patients with a mean bothersomeness/disturbance rating of ≥ 5 (scale: 0–10, 0: not at all bothersome/disturbing; 10: extremely bothersome/disturbing).

**Results:**

Fifteen patients were interviewed (median [range] age: 57 [28–78] years; Pi*ZZ, *n* = 12; Pi*MZ, *n* = 3). Of 41 signs and/or symptoms, the most salient were fatigue/tiredness, respiratory infections, shortness of breath, confusion/difficulty concentrating, and edema. Highly salient signs and/or symptoms were abdominal swelling, acid reflux, sleep disturbance, vomiting, abdominal pain/tenderness, itchiness, and back pain. Of 16 impacts, the most salient were on work and employment, leisure activities, and relationships. Impacts on mobility were highly salient.

**Conclusion:**

Several concepts were frequently reported as moderately/highly bothersome/disturbing. Further investigation of the experience of patients with AATD-LD in a large, diverse population across all fibrosis stages and genotypes is warranted. Clinical outcome assessments that capture salient concepts are needed.

**Supplementary Information:**

The online version contains supplementary material available at 10.1007/s11136-025-03926-x.

## Plain English summary

Alpha-1 antitrypsin deficiency (AATD) is an inherited condition that can cause liver and/or lung disease. A change in a single gene creates an abnormal protein in the liver. Individuals who have two copies of the abnormal gene are protease inhibitor (Pi) ZZ and those with one copy can be Pi*MZ or Pi*SZ. Researchers wanted to know more about how liver disease in AATD (AATD-LD) affects people’s lives. They did this by gathering information on the experiences of patients with AATD-LD from peer-reviewed articles, patient blog posts, and interviews with doctors, and then interviews with 15 US adults with AATD-LD aged 28–78 years. Overall, 41 signs/symptoms were identified and grouped based on their frequency and how much bother they cause. The most frequent and bothersome signs/symptoms were fatigue, respiratory infections, shortness of breath, confusion/difficulty concentrating, and swelling. Other notable signs/symptoms were abdominal swelling, acid reflux, sleep disturbance, vomiting, abdominal pain/tenderness, itchiness, and back pain. The study also identified 16 areas of life affected by AATD-LD; the most affected areas were work/employment, leisure activities, and personal relationships. Mobility was also notably affected. This study focused on a small number of patients and many signs/symptoms were noted in earlier stages of liver disease. These findings highlight the need for additional research in a larger and more diverse group of patients to better understand the experiences of patients with AATD-LD. The development of tools that can accurately measure the experiences of patients with AATD-LD is crucial for improving care.

## Introduction

Alpha-1 antitrypsin deficiency (AATD) is an autosomal codominant genetic condition that manifests clinically as liver and/or lung disease in children and adults [1–4]. AATD is caused by genetic mutations in *SERPINA1*, which encodes alpha-1 antitrypsin (AAT), a serine protease inhibitor (Pi) synthesized mainly in hepatocytes that functions primarily to maintain the protease–antiprotease balance in the lungs [5–8]. Many *SERPINA1* variants exist with variable effects, with Pi*Z and Pi*S being most commonly associated with clinical cases [9, 10]. The homozygous Pi*ZZ genotype carries the greatest risk of AATD-associated liver disease (AATD-LD), followed by the Pi*SZ genotype [11, 12]. The Pi*MZ genotype increases susceptibility to liver injury and promotes the progression of other chronic liver diseases, while the Pi*SS genotype is not associated with hepatic abnormalities [11, 12]. In a large systematic assessment of liver disease burden in a multinational cohort of 403 patients with the Pi*ZZ genotype, the prevalence of significant and advanced liver fibrosis was 20–36% and 5–26%, respectively [13].

Severe AATD manifests clinically as pulmonary dysfunction (asthma, bronchiectasis, chronic bronchitis, chronic obstructive pulmonary disease [COPD], or emphysema) and/or hepatic dysfunction (fibrosis, cirrhosis, hepatitis, hepatocarcinoma, or liver failure) in affected individuals from infancy to adulthood [2, 14]. While previous studies of the quality-of-life (QoL) burden of AATD have focused on AATD-associated lung disease [15], there is limited real-world evidence on the experience of patients with AATD-LD, which remains underdiagnosed owing to its variable and nonspecific clinical presentation and often asymptomatic course until decompensated cirrhosis occurs [16–18]. Individuals with AATD-LD may experience lengthy delays and visit several physicians before receiving a definitive diagnosis; the average delay between the onset of symptoms and diagnosis of AATD-LD exceeds 5 years [19–23]. There are currently no specific treatments for AATD-LD, and liver transplantation is the only way to resolve advanced liver cirrhosis [9, 24]. There are validated patient-reported tools for the assessment of QoL in patients with liver disease, including disease-specific tools such as Liver Disease Quality of Life (LDQOL) and Chronic Liver Disease Questionnaire (CLDQ), and generic tools such as Short-form Survey (SF-36), Sickness Impact Profile (SIP), and Nottingham Health Profile (NHP) [25]. However, these tools have not been used to assess the burden of liver disease in patients with AATD. Therefore, to address this clear unmet need to better understand the experiences of patients with AATD-LD, this qualitative study aimed to evaluate the experiences of patients with AATD-LD and a Pi*ZZ or Pi*MZ genotype in terms of the signs and/or symptoms they experience and their impacts, as well as to understand other issues such as barriers to diagnosis and care and available treatments.

## Methods

### Study design overview

**Fig. 1:**
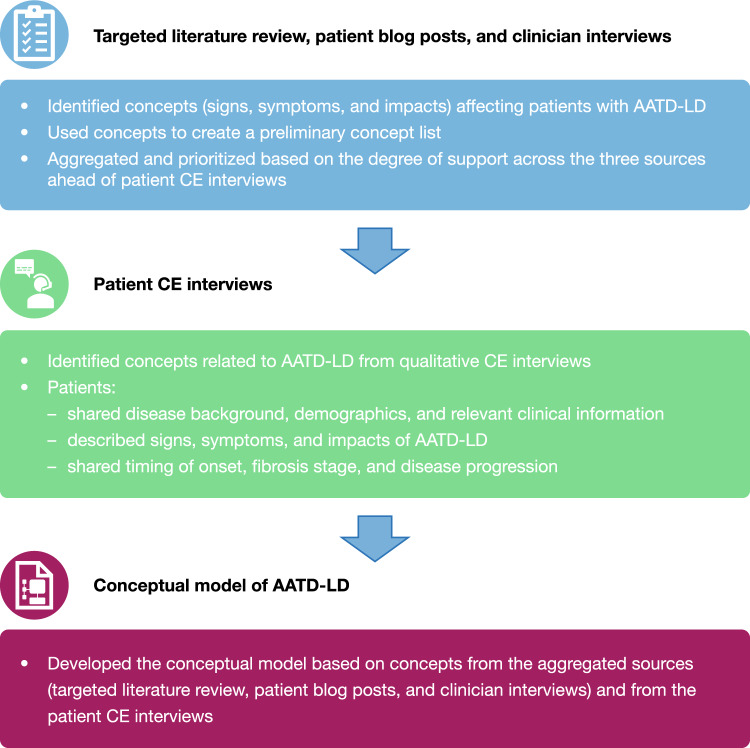
An overview of the study design. *AATD-LD* alpha-1 antitrypsin deficiency-associated liver disease, *CE* concept elicitation

This was a qualitative, cross-sectional, noninterventional, concept elicitation (CE) study. A preliminary concept list was developed from a targeted literature review (TLR), patient blog posts, and clinician interviews to identify “concepts” (signs and/or symptoms, and impacts) affecting patients with AATD-LD, which were aggregated and prioritized based on the degree of support across the three sources ahead of the patient CE interviews. During the interviews, patients shared their disease background, demographics and relevant clinical information, described their experience of the signs and/or symptoms and impacts of AATD-LD, and provided the timing of their symptom onset, their fibrosis stage, and indications of disease progression. A conceptual model was developed based on concepts from the aggregated sources and from the patient CE interviews. An overview of the study design is shown in Fig. 1.

### Development of the preliminary concept list

A TLR was conducted that focused broadly on signs and/or symptoms, and impacts reported by patients with a wide range of chronic liver diseases, including hepatitis C, hepatitis B, metabolic dysfunction-associated steatotic liver disease, metabolic dysfunction-associated steatohepatitis, and AATD. The TLR identified fatigue, nausea, vomiting, and abdominal pain as the most commonly reported symptoms of chronic liver disease, and identified a range of emotional, social, and physical impacts. No specific AATD-LD-related signs and/or symptoms were identified.

Following the TLR, a search of blog posts from patients with AATD-LD was conducted using general search engines (Google and Bing), AATD resource/patient support websites, and websites for AATD patient advocacy groups (Alpha-1 Foundation, Alpha-1 UK Support Group, and Alpha-1 Global). In total, 11 posts from 11 unique patients with AATD-LD were identified (see Online Resource 1, Supplementary Table S1).

To enrich data gathered from the TLR and patient blog posts, interviews were conducted with five clinicians (two gastroenterologists, two pulmonologists, and one hepatologist) who had experience in managing/treating a combined total of 212 patients with AATD-LD (see Online Resource 1, Supplementary Table S2). The clinicians discussed approaches to diagnosis and staging, disease management and progression, clinical presentation, and their perspectives of patients’ experience with AATD-LD.

Insights from each phase of research—the TLR, patient blog posts, and clinician interviews—were taken into consideration when developing a preliminary concept list to discuss with patients during the CE interviews (see Online Resource 1, Supplementary Tables S3 and S4). Signs and/or symptoms identified from at least two of the three sources, or from one of the sources and also reported as appearing earlier than fibrosis stage F4, were included in the preliminary concept list. Impacts identified from two or more sources were also included in the preliminary concept list. Within the preliminary concept list, signs and/or symptoms identified from three sources, those reported as appearing earlier than fibrosis stage F4, and one symptom (muscle weakness) that had particularly strong support in the patient blog search, were classified as “highest priority” for further probing in the CE interviews. Impacts identified from three sources were classified as “highest priority” for further probing.

### Patient recruitment and eligibility criteria for the patient CE interviews

Patients who met the defined eligibility criteria (see Online Resource 1, Supplementary Table S5) were recruited for the CE interviews by Global Perspectives, a recruitment and patient engagement agency, and from an academic medical center in the Midwest of the USA interested in recommending their patients for participation in the study. Global Perspectives identified potential patients via an existing database of individuals who expressed interest in participating in the study and had previously shared their AATD diagnosis. In addition to identification of individuals via the database, Global Perspectives engaged in outreach efforts with patient advocacy groups, social media groups, and Alpha-1 Foundation clinical resource centers. The participating academic medical center shared an informational flyer with eligible patients; the flyer included a link to access and complete the online consent form and screener. Eligible individuals were contacted to participate in the scheduled telephone interview.

A physician-confirmed diagnosis form including fibrosis stage and genotype information was collected for all patients, and fibrosis stage and genotype were determined based on this confirmation (see Online Resource 1, Supplementary Material S6). Eligible patients were English-speaking, US adults (aged 18 years or older) diagnosed with AATD-LD with or without lung involvement, a Pi*ZZ, Pi*SZ, or Pi*MZ genotype, METAVIR fibrosis stage F2–F4, and without liver cancer. Originally, the study was planned to recruit only patients who had AATD-LD with a Pi*ZZ genotype. However, owing to patient recruitment challenges, the eligibility criteria were broadened to include patients with Pi*SZ or Pi*MZ genotypes, given that these patients also experience liver disease.

### Patient CE interviews

Between August 1 and November 2, 2022, patients were interviewed one-to-one by a third-party research organization via telephone and, with the patients’ permission, the interviews were recorded. The interviews lasted approximately 60 min. The interviewers followed a central Institutional Review Board-approved discussion guide to inform the structure and flow of the conversations. The study involved up to three moderators who were trained in interviewing patients for CE and cognitive interviews. The Project Director provided oversight and direction to the study team and ensured that all research procedures were implemented as outlined in the protocol.

Patients were asked to share their background and demographic information, relevant clinical information including any comorbidities, the date of their AATD diagnosis and whether they had a diagnosis of liver disease only or both liver and lung disease diagnoses, receipt of AAT augmentation therapy, fibrosis stage and genotype, and the specialty of the clinician who made the diagnosis and/or was managing the condition. Patients then described the signs and/or symptoms, and impacts they experienced that were thought to be due to their AATD-LD. The interviewers probed on the highest priority concepts from the preliminary concept list to determine which of the concepts were relevant. Details such as severity, duration, frequency, triggers, and coping mechanisms were also discussed. Ratings were collected to determine the degree of bothersomeness or disturbance for signs and/or symptoms and impacts, respectively. Interviewers probed for the timing of disease onset and inquired about patients’ fibrosis stage (in addition to information obtained from their confirmation of diagnosis form) and disease progression.

Qualitative data from the patient CE interviews were analyzed using both deductive and inductive coding techniques. A deductive coding approach allowed researchers to apply findings from previous “waves” of interviews and continuously update the codebook, which was developed prior to the commencement of coding based on other sources, including the targeted literature review, patient blog post search, and clinician interviews. Inductive coding is a technique where codes are derived from the data as concepts and ideas naturally emerge. This combined approach facilitated analysis of concept frequencies and saturation, while also providing an opportunity to thematically analyze new concepts and themes as they emerged.

Each transcript was coded by one coder. An experienced coding lead provided oversight of the coding process and closely reviewed 20% of transcripts to identify any discrepancies and ensure consistency and accuracy of coding. After coding was completed, data were reviewed by the coding lead in collaboration with the coder to ensure that frequency counts, codes for saturation analysis, and overall qualitative coding were accurate. After data were cleaned and any inaccuracies were corrected by the coding lead in collaboration with the coder, coding outputs were generated in MAXQDA (VERBI GmbH, Berlin, Germany), a qualitative analysis software, for analysis and reporting.

### Conceptual model and saturation

Based on the aggregated sources (TLR, patient blog posts, and clinician interviews) and patient CE interviews, a conceptual model of AATD-LD was developed. Concepts were considered as “most salient” if reported by ≥ 50% of patients (*n* = 8) with a mean bothersomeness/disturbance rating of  ≥ 5, “highly salient” if reported by > 5 and < 8 patients with a mean bothersomeness/disturbance rating of  ≥ 5 on a scale of 0–10 (0 as not at all bothersome or disturbing; 10 as extremely bothersome or disturbing). Concepts were mapped to a saliency grid to visualize and characterize relative saliency in the context of all signs and/or symptoms and impacts reported.

To determine concept saturation relating to signs and/or symptoms, that is, confirmation that no new concepts were emerging, interviews were conducted in three waves involving five patients each. This sequencing allowed for minor modifications of the discussion guide and inclusion of new concepts. Transcripts were organized chronologically and then grouped according to waves. Codes were derived and compared across all three waves of interviews. Concept saturation was not evaluated for impacts because signs and/or symptoms were the primary focus of the study. Additionally, not every impact was discussed with every participant because some impacts did not arise until later in the interviews. Finally, the wide range and number of signs and/or symptoms reported by participants did not allow time for an extensive discussion of impacts.

## Results

### Demographics and baseline characteristics

The CE interviews were conducted with 15 patients (median age 57 years [range 28–78 years]), of whom eight were male (53.3%), 12 had a Pi*ZZ genotype (80.0%), eight reported having both liver and lung involvement (53.3%), and three reported that they had previously received AAT augmentation therapy (20.0%; Table 1). Five patients were enrolled from the recruitment/patient engagement agency and 10 patients were enrolled from the academic medical center.

**Table 1:** Patient demographics and baseline characteristics

Characteristics	Patients with AATD-LD (*N* = 15)
*Age, years*	
Median (range)	57 (28–78)
*Sex, n (%)*	
Male	8 (53.3)
*Time since AATD diagnosis, years*	
Median (range)	12 (< 1–35)
*Genotype, n (%)*	
Pi*ZZ	12 (80.0)
Pi*MZ	3 (20.0)
*Fibrosis stage (METAVIR), n (%)*	
F2	6 (40.0)
F3	1 (6.7)
F4	8 (53.3)
*Diagnosis, n (%)*	
Liver and lung	8 (53.3)
Liver only	7 (46.7)
*Transplantation, n (%)*^*a*^	
Liver	3 (20.0)
Lung	1 (6.7)
No transplant	11 (73.3)
*Receipt of AAT augmentation therapy, n (%)*	
Yes	3 (20.0)

*AAT* alpha-1 antitrypsin, *AATD* alpha-1 antitrypsin deficiency, *AATD-LD* alpha-1 antitrypsin deficiency-associated liver disease, *METAVIR* meta-analysis of histological data in viral hepatitis, *Pi* protease inhibitor

^a^Patients who had undergone liver or lung transplantation reported their signs and/or symptoms as well as fibrosis stage prior to the transplantation

### Signs and/or symptoms and impacts

**Fig. 2:**
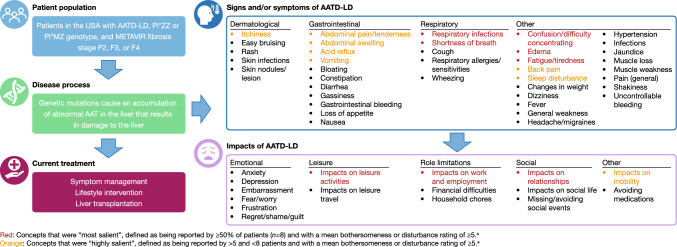
Conceptual model of AATD-LD. ^a^Mean bothersomeness or disturbance ratings were on a scale of 0–10 (0 as not at all bothersome or disturbing; 10 as extremely bothersome or disturbing). Two signs and/or symptoms were excluded from the model owing to the lack of a clear definition (“other respiratory problems” and “other digestive problems”). *AAT* alpha-1 antitrypsin, *AATD-LD* alpha-1 antitrypsin deficiency-associated liver disease, *METAVIR* meta-analysis of histological data in viral hepatitis, *Pi* protease inhibitor

**Fig. 3:**
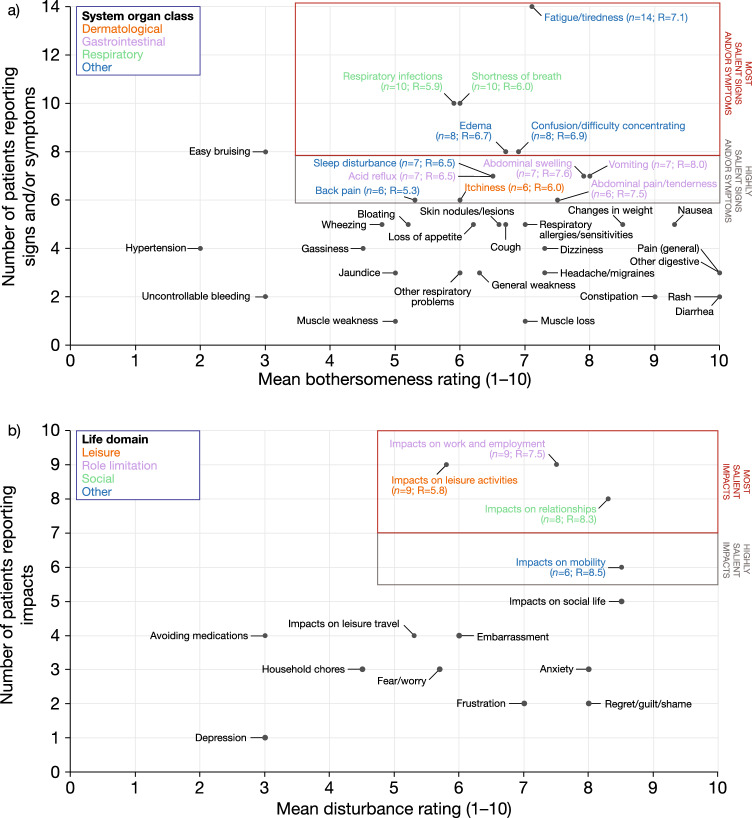
Saliency map of **a** signs and/or symptoms and **b** impacts reported by patients who had AATD-LD with a Pi*ZZ or Pi*MZ genotype (*N* = 15). Concepts assigned as “most salient” were defined as being reported by ≥ 50% of patients (*n* = 8), with a mean bothersomeness or disturbance rating of ≥ 5; concepts assigned as “highly salient” were defined as being reported by > 5 and < 8 patients, with a mean bothersomeness or disturbance rating of ≥ 5 on a scale of 0–10 (0 as not at all bothersome or disturbing; 10 as extremely bothersome or disturbing). The number of patients and mean ratings are displayed only for concepts considered to be “most salient” or “highly salient”. Salient concepts are grouped according to system organ class or life domain. For signs and/or symptoms such as fever, gastrointestinal bleeding, infections, skin infections, and shakiness, the mean bothersomeness ratings are not shown. *AATD-LD* alpha-1 antitrypsin deficiency-associated liver disease, *Pi* protease inhibitor, *R* rating

The CE interviews identified 41 signs and/or symptoms and 16 impacts (Fig. 2). Five signs and/or symptoms were reported as “most salient”: fatigue/tiredness (*n* = 14), respiratory infections (*n* = 10), shortness of breath (*n* = 10), confusion/difficulty concentrating (*n* = 8), and edema (*n* = 8). Seven additional signs and/or symptoms were identified as “highly salient”: abdominal swelling (*n* = 7), acid reflux (*n* = 7), sleep disturbance (*n* = 7), vomiting (*n* = 7), abdominal pain/tenderness (*n* = 6), itchiness (*n* = 6), and back pain (*n* = 6). The saliency map (Fig. 3a) highlights the degree to which fatigue/tiredness—and to a lesser extent, shortness of breath, respiratory infections, confusion/difficulty concentrating, and edema—are separated from the other salient signs and/or symptoms reported. It also highlights the relative importance of gastrointestinal symptoms, such as abdominal swelling, acid reflux, vomiting, and abdominal pain/tenderness, as well as dermatological symptoms (itchiness). The mean (standard deviation [SD]) number of signs and/or symptoms was 14.3 (7.4) in seven patients with F2 or F3 fibrosis and 11.0 (4.1) in eight patients with F4 fibrosis. Patients with fibrosis stage F2 or F3 reported higher mean bothersomeness ratings than patients with fibrosis stage F4 for abdominal pain/tenderness, shortness of breath, sleep disturbance, and itchiness. The mean (SD) number of signs and/or symptoms was 13.4 (5.4) in seven patients with liver and lung involvement and 11.6 (6.7) in eight patients with only liver involvement.

Three impacts were identified as “most salient”: impacts on work and employment (*n* = 9), leisure activities (*n* = 9), and relationships (*n* = 8). One additional impact was identified as “highly salient”: impacts on mobility (*n* = 6). The second saliency map (Fig. 3b) highlights the degree to which impacts on work and employment, leisure activities, and relationships were separated from the other salient impacts reported. In addition, this saliency map underscores the ways in which AATD-LD impairs patients’ abilities to remain socially and professionally active and physically active/mobile. Furthermore, emotional impacts, such as anxiety, fear/worry, frustration, and depression, in aggregate, were relatively prominent and disturbing (with a mean disturbance rating of 6.3), although each individual impact was reported by relatively few patients.

### Saturation of signs and/or symptoms

All but five signs and/or symptoms (fever, muscle loss, muscle weakness, shakiness, and uncontrollable bleeding) were mentioned by patients in the first or second wave of interviews. The most salient concepts were fully elicited; however, saturation was not fully achieved because several less salient concepts arose for the first time in the last wave of interviews.

### Patient experiences with AATD-LD

**Fig. 4:**
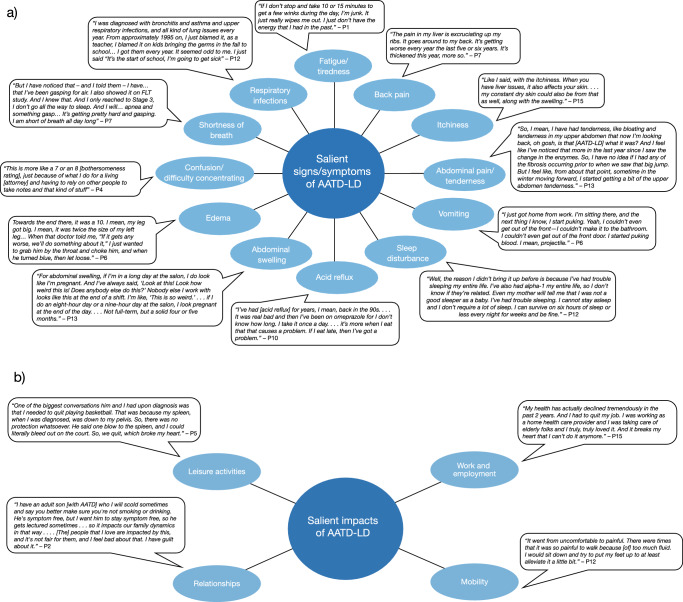
Patient experience of **a** signs and/or symptoms and **b** impacts of AATD-LD. *AATD-LD* alpha-1 antitrypsin deficiency-associated liver disease, *P* patient

To further highlight the varied patient experiences associated with AATD-LD, patients were asked to share their journey with the disease and their experiences with individual signs and/or symptoms and impacts (Fig. 4a, b). Many patients complained of delayed diagnoses and significant barriers in accessing care because of their perception of a clinician’s lack of knowledge about AATD-LD or their perceived unwillingness to take the symptoms seriously. Many patients described repeated visits to multiple clinicians before discovering the underlying cause of their symptoms. Some patients also recounted their experiences with clinicians whom they believed were unwilling to listen to their concerns because of a belief that their liver abnormalities may be due to alcohol misuse. Nearly all patients expressed their frustration at the lack of treatment options available for AATD-LD. Other than liver transplantation in patients with severe cirrhosis and AAT augmentation therapies in patients with concomitant lung disease, patients reported being offered few treatment options other than lifestyle modifications such as weight loss, dietary changes, and avoidance of alcohol and tobacco. Despite these challenges, many patients maintained a positive outlook, and expressed a determination to proactively manage their symptoms and minimize their life impacts.

## Discussion

This small sample of adults with AATD-LD and primarily a Pi*ZZ genotype were highly symptomatic. Several symptom categories—including fatigue/tiredness, respiratory, gastrointestinal, and dermatological—were the most frequently reported and were often characterized by patients as being very bothersome. In addition, the experience of patients with AATD-LD varied, with fatigue/tiredness being the only sign/symptom reported by more than two-thirds of the patient sample. This broadly aligns with the literature on patients with chronic liver disease, for whom fatigue is the most commonly reported symptom, with a prevalence of 35–85% [26]. Moreover, in this patient sample, impacts on many aspects of daily living were noted, particularly physical, social, and emotional categories, all of which have the potential to have a profound toll on patients’ health-related QoL.

A number of signs and/or symptoms, and impacts that were not identified via the TLR, search of patient blog posts, or clinician interviews were newly elicited via the patient CE interviews. These signs and/or symptoms were back pain, changes in weight, constipation, diarrhea, fever, gassiness, headaches/migraines, muscle loss, pain, rash, respiratory infections, respiratory allergies/sensitivities, shakiness, skin infections, skin nodules/lesions, and uncontrollable bleeding. The additional impacts identified in the CE interviews were avoiding medications, embarrassment, financial difficulties, frustration, missing/avoiding social events, and regret/shame/guilt. The additional signs and/or symptoms, and impacts elicited via the patient CE interviews show that the literature and other sources may not fully capture the experiences of patients with AATD-LD. The open-ended, conversational format of the patient CE interviews may have facilitated the sharing of additional experiences than what was reported in blog posts, in the literature, or by clinicians, further highlighting the value of patient interviews.

AATD-LD is considered an asymptomatic, silent, progressive disease, particularly in its early stages, with symptoms appearing gradually as fibrosis progresses, potentially leading to end-stage liver disease [3, 24, 27]. However, patients in this study reported signs and/or symptoms at earlier fibrosis stages, such as shortness of breath, sleep disturbance, and itchiness. These observations may suggest that, unlike other chronic liver diseases, patients with AATD-LD may experience early signs and/or symptoms prior to progressing to advanced fibrosis, although some of these can be perceived as nonspecific to the disease. Although these observations are derived from a small patient sample, given the lack of available data on the experience of patients with AATD-LD, we firmly believe that this qualitative study contributes to an improved understanding in this area, which should be built upon in future quantitative studies to support the clinical management of these patients.

Delayed AATD-LD diagnosis is a major source of frustration among patients owing to the nonspecific signs and/or symptoms, or limited knowledge of the disease or perceived beliefs among clinicians. In our study, some patients believed that their clinicians were unwilling to listen to their concerns because of a belief that their liver disease may have been caused by alcohol misuse. This may highlight a need for improved patient–clinician communication. In addition, many patients reported that they believed that delayed diagnoses and barriers to care which they experienced could be attributed to their clinician’s perceived lack of knowledge about AATD-LD or unwillingness to take the symptoms seriously. This may highlight a need for additional clinician education. The lack of treatment options was also a cause of frustration among our CE patient sample. Currently, there are no specific therapies for preventing liver disease progression in AATD-LD, and liver transplantation is the only available curative treatment [18, 24]. Treatment is limited to lifestyle modifications, such as nutritional management, prevention of obesity, and limiting alcohol intake [18, 28].

This study has several limitations. First, a relatively small number of patients were interviewed who were enrolled from a recruitment/patient engagement agency (*n* = 5 patients) and an academic medical center (*n* = 10 patients) in the USA, which may have been a source of bias. Although patients may have been enrolled from various locations within the USA, the generalizability of our findings to the wider population of individuals with AATD-LD is limited, and the small sample size limits our ability to draw definitive conclusions. Second, patients were not always able to indicate their fibrosis stage or provide detailed information about when signs and/or symptoms, and impacts emerged, thus affecting the precision of their reporting on their own experience. Third, patients were asked during their CE interviews whether they had received a diagnosis of lung disease, but the diagnosis was not confirmed by a physician. Therefore, some patients may have inaccurately reported the presence or absence of lung disease. Fourth, owing to patient recruitment challenges, the study deviated from the original plan of exclusively recruiting patients with a Pi*ZZ genotype by also including patients with a Pi*MZ genotype, whose experience with AATD-LD may differ. Further research with a larger, more geographically representative sample across all verified fibrosis stages is warranted. Fifth, saturation was not fully achieved because several less salient concepts arose for the first time in the last wave of interviews. This is not unexpected in a small population of patients with a rare disease associated with a heterogenous symptom set. Finally, it was not possible to isolate the burden attributed specifically to AATD-LD from the burden associated with comorbidities and environmental factors; nevertheless, this study sought to gain preliminary insights into the burden of AATD-LD and identify the patient population of interest for future quantitative studies.

Further research is needed to determine which signs and/or symptoms, and impacts remain salient in a broader patient population, how the signs, symptoms, and impacts change over time and with disease progression, and which signs and/or symptoms are specific to AATD-LD. Moreover, the heterogeneity of the patient experience observed in our study may also have implications for endpoint selection and measurement in clinical trials. Consideration of clinical outcome assessments that are broad enough to capture the “most salient” and “highly salient” concepts defined in this study is warranted to appropriately assess the nuances of each patient’s experience.

## Conclusions

To the best of our knowledge, this is the first qualitative study to evaluate the experience of patients living with AATD-LD, including an assessment of signs and/or symptoms of the disease and their impacts. The study revealed a heterogeneous sample of patients living with a range of bothersome signs and/or symptoms, and disturbing impacts on daily life.

## Supplementary Information

Below is the link to the electronic supplementary material.

Supplementary file (DOCX 130 KB)

## Data Availability

The datasets generated and/or analyzed during the current study are available from Takeda, with reasonable request to the corresponding author. However, restrictions apply to the availability of these data to protect patient privacy.
