# Supplementary material for: Patient experience of alpha-1 antitrypsin deficiency-associated liver disease: a qualitative study
Source: Qual Life Res. 2025 Mar 13;34(6):1823–32. doi: 10.1007/s11136-025-03926-x (PMC12119735; doi:10.1007/s11136-025-03926-x)
Supplement: Supplementary file 1 — Supplementary file (DOCX 130 KB) [file 11136_2025_3926_MOESM1_ESM.docx]

Patient experience of alpha-1 antitrypsin deficiency-associated liver disease: a qualitative study

***Quality of Life Research***

Virginia C. Clark **·** Suna Park **·** Robert Krupnick **·** Nicole Sparling **·** Jason Ritchie **·** Chitra Karki **·** Justin A. Reynolds

Corresponding author: Chitra Karki, 40 Landsdowne Street, Cambridge, MA 02139, USA; email: [chitra.karki@takeda.com](mailto:chitra.karki@takeda.com)

# Supporting information

## Supplementary Table S1 Patient blog posts

| **Title** | **Link** |
| --- | --- |
| —^a^ | [https://www.alpha1.org/rare-disease-day](https://www.alpha1.org/rare-disease-day/) |
| —^a^ | <https://www.alpha1.org/rare-disease-day> |
| —^a^ | <https://www.alpha1.org/rare-disease-day> |
| My Alpha 1 Story | <https://www.diseasemaps.org/alpha-1-antitrypsin-deficiency/story/773/> |
| I Have What? | <https://friendsoftheslulc.org/wp-content/uploads/2012/06/Lindsays-story.pdf> |
| Being an Alpha-1 Carrier | <https://www.alpha1.ie/being-an-alpha-1-carrier-mary-s-story/> |
| — | <https://liverfoundation.org/about-your-liver/patient-stories/linda-k/> |
| Matt’s Story: A Three Organ Simultaneous Transplant and a New Lease on Life | <https://www.donatelifenc.org/blog/matts-story-three-organ-simultaneous-transplant-and-new-lease-life> |
| Linda’s Story | <http://www.uwmedicine.org/sites/stevie/files/2021-05/LDLT-Kathryn-and-Linda.pdf> |
| How BMX Triggered My Liver Disease Discovery | <https://childliverdisease.org/how-bmx-triggered-my-liver-disease-discovery/> |
| Warning: She is in a Bad Mood | <https://www.fattyliverfoundation.org/patient_stories?page=4> |

^a^For the first three patient blog posts, only responses from individuals with AATD indicating liver involvement and reporting signs and/or symptoms and impacts were included. Referenced posts were accessed on August 3, 2023

*AATD* alpha-1 antitrypsin deficiency

## Supplementary Table S2 Clinician interview sample (*N*=5)

| **Specialty** | **Years in practice** | **Practice setting** | **Number of patients with AATD-LD** |
| --- | --- | --- | --- |
| Gastroenterology | 11 | Community-based hospital | 12 |
| Gastroenterology | 10 | Hospital-based office practice | 50 |
| Pulmonology | 20 | Private practice | 20 |
| Pulmonology | 20 | Academic medical center | 30 |
| Hepatology | 26 | Academic medical center | 100 |

*AATD-LD* alpha-1 antitrypsin deficiency-associated liver disease

## Supplementary Table S3 Signs and/or symptoms of AATD-LD: preliminary concept list as identified from the TLR, patient blog posts, and clinician interviews

| **TLR** | **Patient blog posts** | **Clinician interviews** | **Concept for inclusion in patient CE interviews** |
| --- | --- | --- | --- |
| ***Energy-related*** | | | |
| Dizziness | + | + | **Dizziness** |
| Energy |  |  |  |
| Exhaustion |  |  |  |
| Fatigue | + | + | **Fatigue*** |
| Somnolence |  |  |  |
| Tiredness |  | + | Tiredness |
| Vitality |  |  |  |
| Weakness |  | + | Weakness |
| ***Gastrointestinal-related*** | | | |
|  |  | Abdominal discomfort | **Abdominal discomfort*** |
| Abdominal pain |  | + | **Abdominal pain*** |
| Abdominal swelling | + | + | **Abdominal swelling** |
|  |  | Abdominal tenderness | **Abdominal tenderness*** |
| Acid reflux | + | +/- | Acid reflux |
|  |  | Bloated feeling | **Bloated feeling*** |
| Constipation |  |  |  |
| Diarrhea |  |  |  |
| Gastrointestinal bleeding |  | + | Gastrointestinal bleeding |
| Nausea/vomiting |  | + | **Nausea/vomiting*** |
| ***Skin-related*** | | | |
| Bruising |  | + | **Easy bruising*** |
| Dermatitis |  |  |  |
| Itching |  | + | Itching |
| Jaundice | + | + | **Jaundice*** |
| Skin disorders |  | + | Skin disorders |
| Skin hyperpigmentation |  |  |  |
| ***Cognition-related*** | | | |
| Ability to concentrate |  | + | Difficulty concentrating |
| Cognitive functioning |  | Confusion | Confusion |
| Loss of memory |  |  |  |
| Loss of attention |  |  |  |
| ***Respiratory-related*** | | | |
|  | Cough | +/- | Cough |
| Dyspnea | Shortness of breath | +/- | Shortness of breath |
|  | Wheezing | +/- | Wheezing |
| ***Other*** | | | |
| Anorexia |  |  |  |
|  |  | Bleeding | Bleeding |
| Dry mouth |  |  |  |
|  | Edema | + | Edema |
|  | Enlarged spleen | + | Enlarged spleen |
| Fever |  |  |  |
|  | Headache | + | Headache |
|  | Hypertension | +/- | Hypertension |
|  | Infections | + | Infections |
| Insomnia/sleep |  | + | Sleep disturbances |
| Joint pain |  |  |  |
| Loss of appetite |  | + | **Loss of appetite*** |
|  | Muscle atrophy | + | Muscle loss |
|  | Muscle weakness |  | **Muscle weakness** |
|  | Numbness | +/- | Numbness |
| Pain |  |  |  |
| Sexual function |  | -/+ | Sexual functioning |
| Systemic symptoms |  |  |  |

Note: +, concept may be relevant to patients; +/-, disagreement among clinicians about relevance of concept; *, reported earlier than fibrosis stage F4; , no evidence was available (concept was not mentioned or not asked); signs and/or symptoms in **bold** were classified as ‘highest priority’ for further probing in the patient CE interviews if they had support from all three sources or were reported as appearing earlier than fibrosis stage F4. In addition, one symptom (muscle weakness) was classified as ‘highest priority’ owing to particularly strong support from the search of patient blog posts.

*AATD-LD* alpha-1 antitrypsin deficiency-associated liver disease, *CE* concept elicitation, *TLR* targeted literature review

## Supplementary Table S4 Impacts of AATD-LD: preliminary concept list as identified from the TLR, patient blog posts, and clinician interviews

| **TLR** | **Patient blog post** | **Clinician interviews** | **Concept for inclusion in patient CE interviews** |
| --- | --- | --- | --- |
| ***Emotional*** | | | |
| Altered body image | + | + | **Altered body image** |
| Anxiety | + | + | **Anxiety*** |
| Confidence | + |  | Confidence |
| Depression | + | + | **Depression*** |
| Fear | + | + | **Fear*** |
| Frustration |  |  |  |
| Irritability |  |  |  |
| Low mood |  |  |  |
|  | Low self-esteem | + | Low self-esteem |
| Sadness |  |  |  |
| Self-consciousness | + |  | Self-consciousness |
|  | Shock | + | Shock |
| Stigma |  |  |  |
| Stress |  |  |  |
| Worry | + | + | **Worry*** |
| ***Physical*** | | | |
| Bending over |  | + | Bending over |
| Disabled | + | + | **Disabled** |
| Reaching |  |  |  |
| Mobility |  | + | Mobility |
| Unable to walk/exercise | + | + | **Leisure activities/ exercise** |
| ***Role*** | | | |
| Activities of daily living | + | + | **Activities of daily living** |
| Household chores | + | + | **Household chores** |
| Self-care |  |  |  |
| Self-management |  |  |  |
| *Social* | | | |
| Family and caregivers | + | + | **Family and caregivers** |
| Friendships |  |  |  |
| Hiding symptoms from others |  |  |  |
| Life choices |  |  |  |
| Quality of social interactions | + | + | **Social life** |
| Relationships with others |  |  |  |
| *Work and employment* | | | |
| Absenteeism | + | + | **Absenteeism** |
| Job loss | + | + | **Job loss** |
| Work productivity | + | + | **Work productivity** |
| ***Other*** | | | |
| Diet/nutrition |  | Malnutrition | Malnutrition |
| ~ | Financial | + | Financial |
| Lack of knowledge about disease |  |  |  |
| Sex life |  | +/- | Sex life |
| Sleep |  | + | Sleep |
| ***Treatment-related*** | | | |
| Access to care |  |  |  |
| Financial |  |  |  |
| Medical adherence |  |  |  |
| Side effects |  |  |  |

Note: +, concept may be relevant to patients; +/-, disagreement among clinicians about relevance of concept; *, reported earlier than F4; , no evidence was available (concept was not mentioned or not asked); impacts in **bold** were classified as ‘highest priority’ for further probing in the patient CE interviews if they had support from all three sources.

*AATD-LD* alpha-1 antitrypsin deficiency-associated liver disease, *CE* concept elicitation, *TLR* targeted literature review

## Supplementary Table S5 Patient eligibility criteria for participation in the CE interviews

| **Inclusion criteria** | **Exclusion criteria** |
| --- | --- |
| - Diagnosis of AATD-LD - Pi*ZZ, Pi*MZ, or Pi*SZ genotype. For patients with comorbid ALD and hepatitis C, only patients with Pi*ZZ genotype were included - 18 years or older - Resident in the continental USA - English-speaking - Willing and able to electronically sign an informed consent or assent form - Willing and able to participate in an approximately 60-minute telephone interview - Access to a telephone, a mobile device, or an internet-connected computer | - History of either significant neurological events or a mental condition rendering the patient unable to understand the nature, scope, and possible consequences of the study - Liver cancer |

*AATD-LD* alpha-1 antitrypsin deficiency-associated liver disease, *ALD* alcoholic liver disease, *CE* concept elicitation, *Pi* protease inhibitor

## Supplementary Material S6 Confirmation of diagnosis form

##
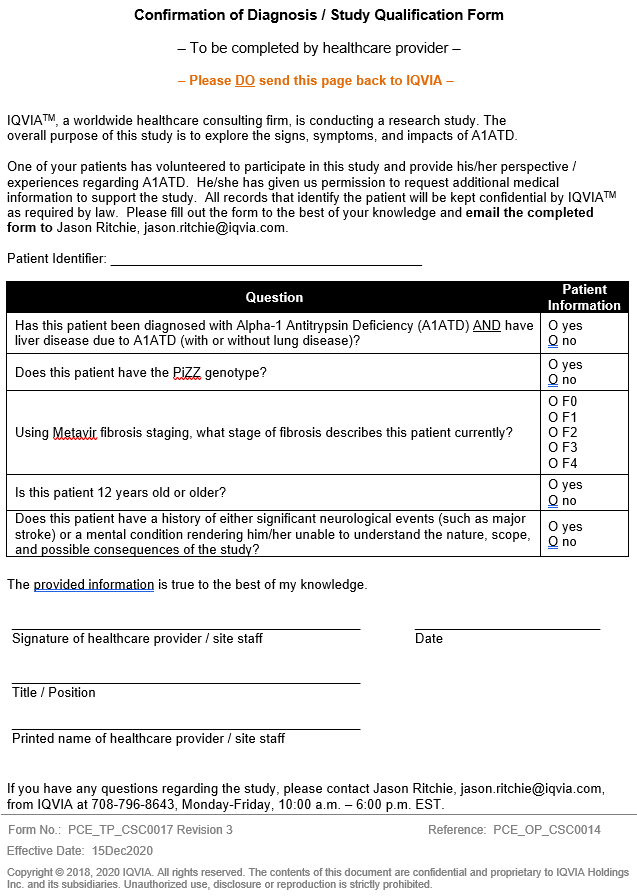


*A1ATD* alpha-1 antitrypsin deficiency, *Metavir* meta-analysis of histological data in viral hepatitis
